# Effect of Si_3_N_4_ Additive on Microstructure and Mechanical Properties of Ti(C,N)-Based Cermet Cutting Tools

**DOI:** 10.3390/ma17112586

**Published:** 2024-05-28

**Authors:** Ali Elgazzar, Sheng-Jian Zhou, Jia-Hu Ouyang, Zi-Jian Peng, Jun-Teng Yao, Zhan-Guo Liu, Yu-Jin Wang, Ya-Ming Wang

**Affiliations:** 1 School of Materials Science and Engineering, Harbin Institute of Technology, Harbin 150001, China; 2 Mechanical Engineering Department, Faculty of Engineering at Shoubra, Benha University, Cairo 11629, Egypt

**Keywords:** Ti(C,N)-based cermets, microstructure, core–rim structure, silicon nitride, mechanical properties, thermal stability

## Abstract

Development of high-performance cutting tool materials is one of the critical parameters enhancing the surface finishing of high-speed machined products. Ti(C,N)-based cermets reinforced with and without different contents of silicon nitride were designed and evaluated to satisfy the requirements. In fact, the effect of silicon nitride addition to Ti(C,N)-based cermet remains unclear. The purpose of this study is to investigate the influence of Si_3_N_4_ additive on microstructure, mechanical properties, and thermal stability of Ti(C,N)-based cermet cutting tools. In the present work, α-Si_3_N_4_ “grade SN-E10” was utilized with various fractions up to 6 wt.% in the designed cermets. A two-step reactive sintering process under vacuum was carried out for the green compact of Ti(C,N)-based cermet samples. The samples with 4 wt.% Si_3_N_4_ have an apparent solid density of about 6.75 g/cm^3^ (relative density of about 98 %); however, the cermet samples with 2 wt.% Si_3_N_4_ exhibit a superior fracture toughness of 10.82 MP${\mathrm{a.m}}^{1/2}$ and a traverse rupture strength of 1425.8 MPa. With an increase in the contents of Si_3_N_4_, the Vickers hardness and fracture toughness of Ti(C,N)-based cermets have an inverse behavior trend. The influence of Si_3_N_4_ addition on thermal stability is clarified to better understand the relationship between thermal stability and mechanical properties of Ti(C,N)-based cermets.

## 1. Introduction

In the field of cutting-edge developments, quality tooling is one of the characteristics required for optimal machining and to assess optimized operations to assure the continued success of brilliant surface roughness [1]. Because finish machining requires cutting tools that are sharp, choosing the right tool is essential. During the machining process, a large amount of removal material was obtained. The main requirement is the existence of a cutting tool with specific properties to support the heat generated during the cutting process [2]. Dry machining requires the selection of a tool material that may preserve hardness at elevated temperatures and withstand extreme conditions, as the process produces higher temperatures when compared with wet cutting, making it significant for effective dry machining [3]. In addition, the selection of proper material for the cutting tool plays a vital and fundamental role during high-speed metal cutting, such as supporting the generated heat, decreasing cutting forces, and boosting abrasion resistance [4]. Cermets are among the most widely used powder metallurgy products globally for manufacturing cutting tool inserts, owing to their superior hardness and toughness compared to other types of cutting materials [5,6].

Ti(C,N)-based cermet is considered as one of the most widespread and commonly used types, similar to other composite materials consisting of interconnected ceramics and metals. Furthermore, it possesses the advantages of both ceramics and metals, characterized by a core/rim structure [7]. The core–rim structure has a significant impact on the fracture toughness, where the rim is made up of different particles, including tungsten carbide (WC), that inhibit grain growth for Ti(C,N) [8]. The properties of cermets depend mainly on the additives, which are utilized to boost their high hardness and wear resistance properties and minimize the limitation of their low fracture toughness [9]. To achieve the desired properties in machining applications, the selection of the reinforcing agent, along with its morphology or orientation, is an extremely crucial parameter. Reinforcements could be utilized in the form of particulate, laminates, fibers, and whiskers [10]. Additives, especially carbon (C) and nitrogen (N), have a significant influence on Ti(C,N)-based cermets. The presence of carbon within a specified range alters the generated phases, with 1–2.5 wt.% representing the ideal level. Beyond this limit, dissociative carbon phases appear, resulting in deteriorated microstructures [9,11]. Nitrogen, in the form of TiN or Ti(C,N), is necessary for mechanical properties, while the addition of TiN promotes enhanced densification, wear resistance, and grain growth stability [5,12]. Ti(C,N)-based cermets are reinforced with various metal carbides to improve particular features [13]. They can influence the solid solution reaction, grain growth, and core/rim structure of Ti(C,N)-based cermets, which will cause a change in the material’s mechanical features [14]. The addition of both WC and Mo_2_C can enhance the wettability between the core and metallic phase and improve sintering properties of Ti(C,N)-based cermets to obtain greater performance during dry machining [15,16,17]. Furthermore, for boosting the thermal shock resistance and hot hardness, NbC plays a crucial role in this issue [13,18]. Moreover, Ni and Co particles are utilized as a metal binder phase, which might influence the toughness of Ti(C,N)-based cermets by compensating for the brittle nature of Ti(C,N) particles [19,20,21]. Maurya et al. [22] focused on producing Co- and Ni-free Ti(C,N)-Fe-based cermets to build green and cost-effective materials that exhibited improved mechanical capabilities at high temperatures compared to typical WC-based cermets. Ti(C,N)-based cermets demonstrated a combination of transgranular, intergranular, deflection, and branching fracture processes, with better fracture toughness found in cermets containing less porosity and more binder content. However, the study does not address the possible obstacles or limitations connected with the industrial-scale manufacturing of Ti(C,N)-FeCrMo-based cermets, which could impair their commercial viability and scalability.

To mitigate the heat generated during the cutting process and enhance the thermal stability of TiCN-based cermet cutting tools, silicon nitride (Si_3_N_4_) may be a considerable additive. Si_3_N_4_ offers a unique combination of properties, including high-temperature hardness and strength, chemical inertness, and exceptional thermal shock resistance [23]. Its high melting point and outstanding thermal shock resistance allow cermets to maintain their hardness and toughness at extreme temperatures. This is particularly significant in high-speed machining operations, where tools are exposed to intense temperatures and thermal cycling [24]. Furthermore, Si_3_N_4_ has the ability to form a solid solution with Ti(C,N), resulting in a finer microstructure and higher mechanical strength. This results in cermets that are more resistant to deformation and can keep their cutting edge for longer periods of time, extending tool life and enhancing machining efficiency [8]. In summary, adding Si_3_N_4_ to Ti(C,N)-based cermets has various benefits, including increased hardness, toughness, and thermal stability. Improvements like these make Si_3_N_4_ a vital component for improving the performance and lifetime of cutting tools and other industrial applications.

Researchers and manufacturers are particularly concerned with preparing very dense tool materials with precise microstructures by selecting a suitable sintering method and optimizing the sintering process. Spark plasma sintering (SPS) [25,26], hot pressing (HP) [27,28], microwave sintering (MS) [29], hot isostatic pressing (HIP) [30], and pressureless vacuum sintering [31] are all examples of common sintering techniques. Reactive sintering is favored over spark plasma sintering (SPS) since the short sintering time required by SPS makes it difficult to obtain a fully developed and timely core/rim structure.

Because the sintering process may produce Ni_3_Si, precise mixing with silicon nitride is required. The high content of Ni_3_Si can have a negative impact on the relative density and wettability of hard-phase Ti(C,N) particles. Furthermore, the further inclusion of nano-α Si_3_N_4_ promotes grain agglomeration [32]. Shanmugavel et al. [33] investigated the effect of Si_3_N_4_ addition on the mechanical characteristics of cermet compositions. The cermet compositions were produced using the spark plasma sintering process, with the additions of 5 wt.% and 10 wt.% Si_3_N_4_. They discovered that the cermet with a 5 wt.% Si_3_N_4_ addition produced the largest increase in hardness and toughness due to the creation of a core–rim structure. Nevertheless, the influence of Si_3_N_4_ on thermal stability and oxidation resistance was not completely explored. Finally, XRD might be used to examine the microstructure and generated phases of the produced samples. However, with the addition of Si_3_N_4_, Ti(C,N)-based cermet samples may contain undissolved WC phases, which can increase the fracture toughness. One of the reasons for the high K_*IC*_ value is the presence of WC, which has a higher elastic modulus than Ti(C,N). The WC phase absorbs energy and keeps cracks from growing [34]. The creation of an oxidation scale, which functions as a protective barrier, isolating the substrate from the surrounding air, is critical for establishing strong oxidation resistance at high temperatures. However, two more forms of stress develop throughout the oxidation process. One is growth stress, which occurs as the oxide scale expands during isothermal oxidation. The other is thermal stress, which is induced by the substrate and oxide scale’s different rates of thermal expansion or contraction [35]. Veerapandian et al. [28] focused on producing Ti(C,N)-based cermets by adding B_4_C by vacuum hot pressing, and evaluated their microstructure, mechanical characteristics, wear behavior, and thermal stability. The microstructural investigation indicated a core–rim structure, with the addition of B_4_C impacting the formation of TiB and TiB_2_ precipitates, which affect particle size and porosity. However, the sintered cermets display lower hardness when annealed, since the creation of TiO_2_ affects the thermal stability. Furthermore, the study did not investigate the long-term stability or performance of cermets under extended wear conditions, which may be critical for practical use in production environments.

According to recent research, a few studies have concentrated on the fabrication of Ti(C, N)-based cermet cutting tool materials by adding silicon nitride. The novelty of this study lies in using a two-step reactive sintering process to investigate the influence of Si_3_N_4_ addition on the mechanical properties of Ti(C,N)-based cermet cutting tools. The objectives of a two-step sintering process involve removing residual oxygen from the adsorbed surface or lattice, increasing mutual diffusion of elements in carbonitrides, and ensuring complete and uniform heating of the samples. This process begins with pre-sintering to create a porous intermediate structure that facilitates the diffusion of reactive species, followed by reactive sintering to induce further densification and chemical interactions between constituent powders. This stage’s reactions can produce new phases, solid solutions, or compounds, all of which have a significant impact on the microstructure and characteristics of the final material. As a result, the two-step reactive sintering process provides increased control over densification, phase composition, and microstructure, allowing for the optimization of mechanical, thermal, and chemical properties, making it ideal for producing advanced materials such as Ti(C,N)-based cermets with Si_3_N_4_ additions. The main objective of the research is to investigate the influence of silicon nitride content on the microstructure, mechanical properties, and thermal stability of Ti(C,N)-based cermet cutting tools. The microstructural characteristics and mechanical properties, including hardness and toughness, are determined through calculations. To study the high temperature resistance, thermal stability analysis was carried out on the sintered samples. The subsequent sections will delve into the methodology, results, and discussion, providing a closer look at the impact of Si_3_N_4_ on the microstructure, mechanical properties, and thermal stability of Ti(C,N)-based cermets.

## 2. Materials and Methods

### 2.1. Starting Raw Powders

The used commercial raw powders were Ti(C_0.5_N_0.5_) (with average particle size of 0.794 ± 0.02 μm), five types of carbides (WC, Mo_2_C, NbC, VC, Cr_3_C_2_) (with average particle size of 0.535 ± 0.0024 μm, 0.73 ± 0.07 μm, 0.706 ± 0.044 μm, 0.23 ± 0.01 μm, 0.837 ± 0.02 μm, respectively), and two types of metal powders (Ni, Co) with average particle size of 1.9 ± 0.04 μm, 0.54 ± 0.04 μm, respectively. Jilin Changyu Tetao New Material Technology Co., Ltd. (Jilin, China), provided powders with purity above 99.9%. In order to study the influence of silicon nitride addition, α-Si_3_N_4_ “grade SN-E10” with average particle size of 0.164 ± 0.005 μm was utilized with various fractions of 0–6 wt.%. An additional 0.5 wt.% of carbon was added to remove the absorbed oxygen, and 3 wt.% of PEG was added as a forming agent.

### 2.2. Sample Preparation

**Figure 1:**
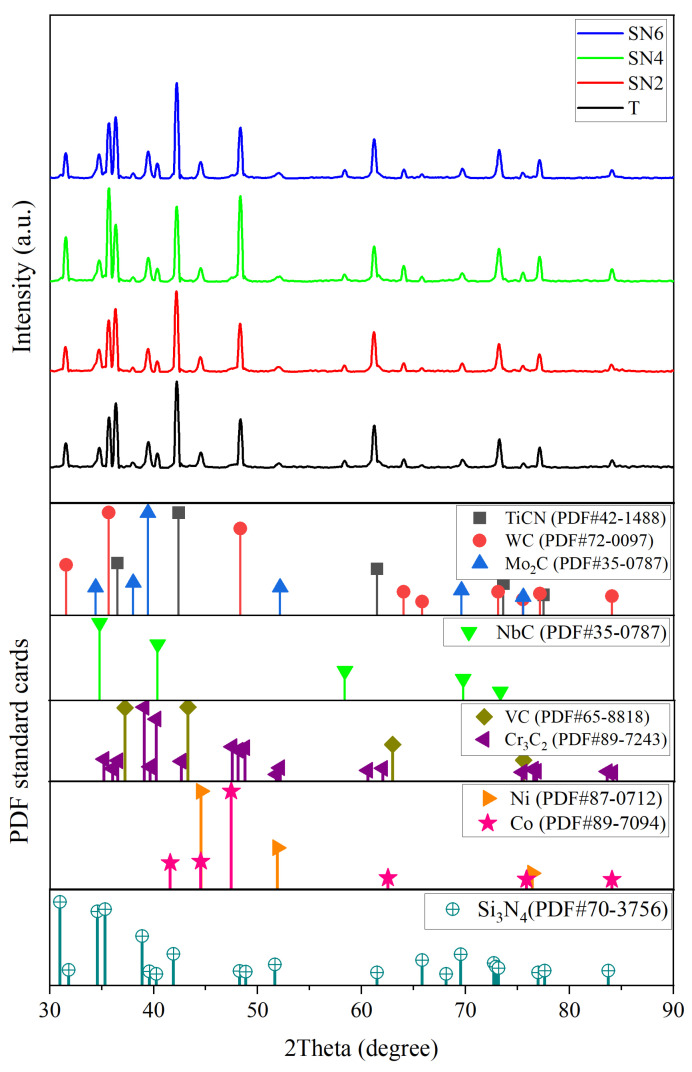
XRD pattern for the milled powders with different contents of Si_3_N_4_.

The initially selected powders were added according to the Table 1’s composition and then wet ball milled for 24 h at a speed of 250 rpm using a stainless steel vial filled in with 5 mm tungsten carbide (WC-Co) balls. The ball-to-powder ratio remained at 10:1 during mechanical milling, where ethanol and water were utilized as milling mediums. The slurry was then dried under vacuum at 90 °C for 18 h. The powders were sieved with a 100-mesh sieve, then manually granulated before being sieved again with a 200-mesh sieve. The patterns obtained from XRD for the dry ball-milled powders are presented in Figure 1. After pressing and forming the milled powder under a pressure of 100 MPa and a holding time of 3 min, the green compact samples were vacuum sintered at 1425 °C for 1 h.

### 2.3. Material Characterization

An X-ray Diffractometer (XRD, X’pert Pro, London, UK) with a monochromatic Cu-K α radiation in a 2θ range of 10–90° was used to identify the phase components of the as-synthesized Ti(C, N)-based cermet powders. The measurements were taken at a scanning rate of 10° min^−1^ and a step size of 0.02°.

A field-emission scanning electron microscope (FESEM, Zeiss Maerlin Compact, Oberkochen, Germany) equipped with energy dispersive X-ray spectroscopy (EDS) for compositional analysis was used to examine the prepared powder morphology, surface morphology, and microstructure of the composites, as well as the morphology of fractured surfaces of the prepared cermets, on diamond-polished and gold-coated faces of different samples.

The particle size of powders was calculated based of the length powder using ImageJ software (free version 3.14), and the data were drawn using Origin. Non-linear curve fitting of the particle size for all raw powders was generated using the following Gaussian formula:

(1) $$y=y_{o}+\frac{A}{w\times \sqrt{\left(\pi /2\right)}}e^{-2\frac{{\left(x-x_{c}\right)}^{2}}{w^{2}}}$$

where *y*, *y_o_* are the independent parameters, *w* is the width of the curve, *A* is the area under the curve, and *X_c_* is the average value of the dependent parameter.

The apparent densities of the cermet specimens were determined using the Archimedes method, following the guidelines outlined in the international standard “ISO18754” [36]. Subsequently, the final relative densities were calculated based on the rule of mixtures. In addition, under the ISO 14705:2016 [37] and ISO 6507-1:2018 [38] procedures, the Vickers hardness tests were performed. A Vickers hardness tester (HVS-30 type) was utilized to measure the hardness under a loading condition of 294.2 N/15 s, in which the following expression was used:

(2) $$H_{v}=1.8544\times 10^{-2}\times \frac{P}{d^{2}}$$

where $H_{v}$ is the Vickers hardness (GPa), *P* is the indentation load (N), *D* is the average indentation diagonal length (mm). Based on the Palmqvist toughness test ISO 28079 [39], another method can be used to calculate the fracture toughness, which is calculated by the length of cracks produced by the hardness test and the hardness value of the material. The average value of 5 to 8 points in the test is taken. The formula for calculating the fracture toughness of cermets “MP${a\;m}^{1/2}$” is:

(3) $$K_{IC}=0.15\sqrt{\frac{HV_{30}}{\Sigma_{i=1}^{4}L_{i}}}$$

where *HV*_30_ and *L_i_* are the Vickers hardness “kg_*f*_/mm^2^” and crack length, respectively under 30 kg load. Based on the standard test method for flexural strength ASTM C1161-13 [40], traverse rupture strength testing was carried out by using a three-point bending stress configuration with a span of 15 mm and a crosshead displacement speed of 0.5 mm/min. Traverse rupture strength was calculated using the following equation:

(4) $$\sigma_{f}=\frac{3PL}{2bh^{2}}$$

where $\sigma_{f}$ is the flexural strength of cermets (MPa), *P* is the fracture load (N), *L* is the span length (mm), *b* is the width of the sample (mm), *h* is the thickness of the specimen (mm).

### 2.4. Thermal Stability

Prior to the oxidation process, Ti(C,N)-based cermets were cut into 5 mm × 6 mm × 10 mm rectangular bars and ground with various grades of SiC abrasive papers and mirror finished with diamond pastes. The thermal stability of Ti(C,N)-based cermets was studied by heat treatment of the samples at 800 °C for 5, 10, and 20 h using a high-temperature resistance heating furnace, followed by furnace cooling. Vickers hardness, by applying a load of 30 kg for a 15 s dwell time, was measured after the oxidation process to identify the influence of Si_3_N_4_. The mass gain was calculated by measuring the mass before and after oxidation.

## 3. Results and Discussion

### 3.1. Powder Characterization

#### 3.1.1. Raw Powders

**Figure 2:**
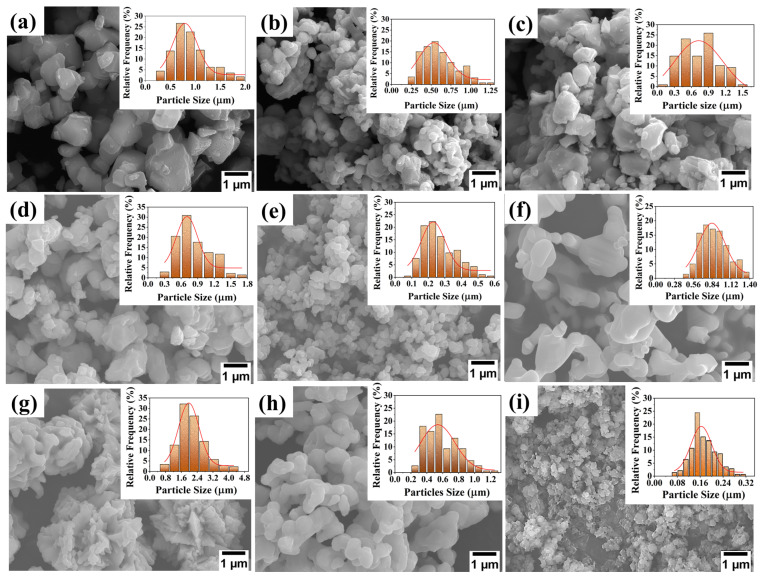
Microscopic morphology and particle size distribution of the initial raw powders: (**a**) Ti(C_0.5_N_0.5_), (**b**) WC, (**c**) Mo_2_C, (**d**) NbC, (**e**) VC, (**f**) Cr_3_C_2_, (**g**) Ni, (**h**) Co, and (**i**) Si_3_N_4_.

Figure 2 shows the microscopic morphology and particle size distribution of raw powders. It can be seen from the figures that the shape of all raw material powders is nearly spherical, the particle size distribution is uniform.

#### 3.1.2. Milled Powders

According to the phase analysis, Figure 1 presents the XRD patterns of the ball-milled powders prior to sintering, confirming the presence of Ti(C,N), WC, Mo_2_C, NbC, VC, Cr_3_C_2_, Ni, Co, and Si_3_N_4_ as starting powders based on the JCPDS numbers 42-1488, 72-0097, 35-0787, 89-3830, 65-8818, 89-7243, 87-0712, 89-7094, and 70-3756, respectively. In addition, no further phases were created. Milling was used to ensure that the particles were mixed uniformly.

### 3.2. As-Sintered Cermet Characterization

**Figure 3:**
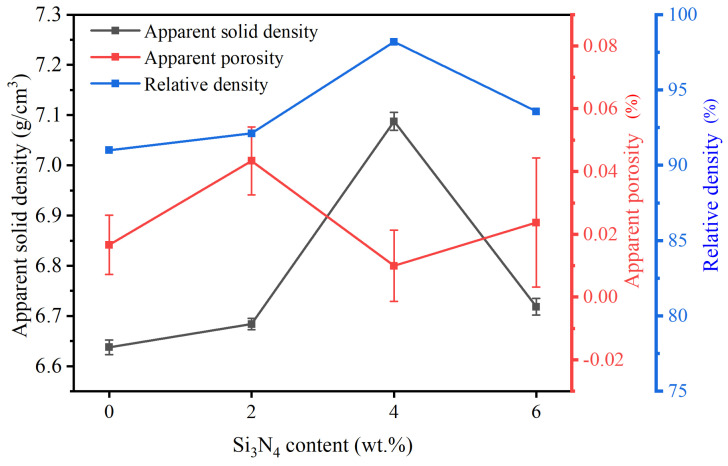
Effect of Si_3_N_4_ contents on Ti(C,N)-based cermet density. Error bars represent the standard deviation (SD).

Figure 3 shows the effect of Si_3_N_4_ contents on the density of the sintered cermet cutting tool. It is shown that as the percentage of silicon nitride addition varies, the apparent solid density and apparent porosity are also changed. A slight increase in density was observed as the content of Si_3_N_4_ was raised from 0 wt.% to 2 wt.%. Additionally, with the increase in silicon nitride content, there was a sharp rise in the value of the apparent solid density from about 6.68 to 7.08 g/cm^3^. However, following its peak, the apparent density subsequently declines to 6.7 g/cm^3^. Within the percentage range of 0.01 to 0.043%, the porosity undergoes regular variations, with slight changes in its magnitude. Because of the variations in the particle size, the addition of Si_3_N_4_ influences the apparent solid density of Ti(C,N)-based cermets. However, excessive Si_3_N_4_ addition reduces the density. Ti(C,N)-based cermet samples with addition of 4 wt.% of Si_3_N_4_ promote the largest relative density of about 98%.

**Figure 4:**
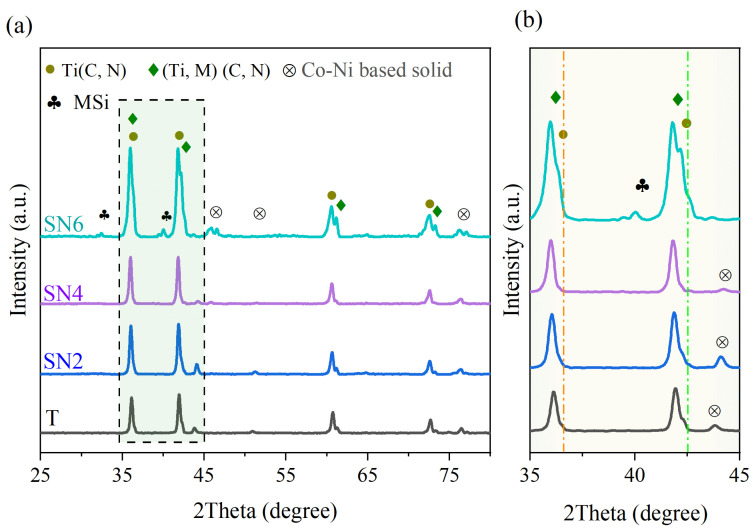
XRD of sintered cermets with the addition of different Si_3_N_4_ contents: (**a**) ranging from 2θ = 25° to 80° and (**b**) enlarged XRD patterns ranging from 2θ = 35° to 45°.

Figure 4 shows the XRD of the sintered sample with different contents of silicon nitride. Studying the several formed phases is essential to recognize the performance of cermets. It can be seen that the phases of the four cermet systems are mainly (Ti, M) (C, N) (M = W, Mo, Nb) solid solution phase. Furthermore, new phases are formed after the sintering process. New phases of Ti_0.8_W_0.2_C and Ti_0.5_Mo_0.5_C with JCPDS numbers 65-7130 and 65-7552, respectively, have developed at the same intensity peak but with slightly different diffraction angles. With the increase in the content of Si_3_N_4_, the overall diffraction peak shifts towards a lower angle side. This may be due to the formation of a new phases with the addition of Si_3_N_4_. It may react with Ti(C,N) and other ingredients and form new phases such as TiSi_2_ and MoSi_2_ (MSi phase at XRD) with JCPDS numbers 89-5588 and 81-2167, respectively. The intensity peaks increase with increasing content of Si_3_N_4_.

### 3.3. Microstructures

**Figure 5:**
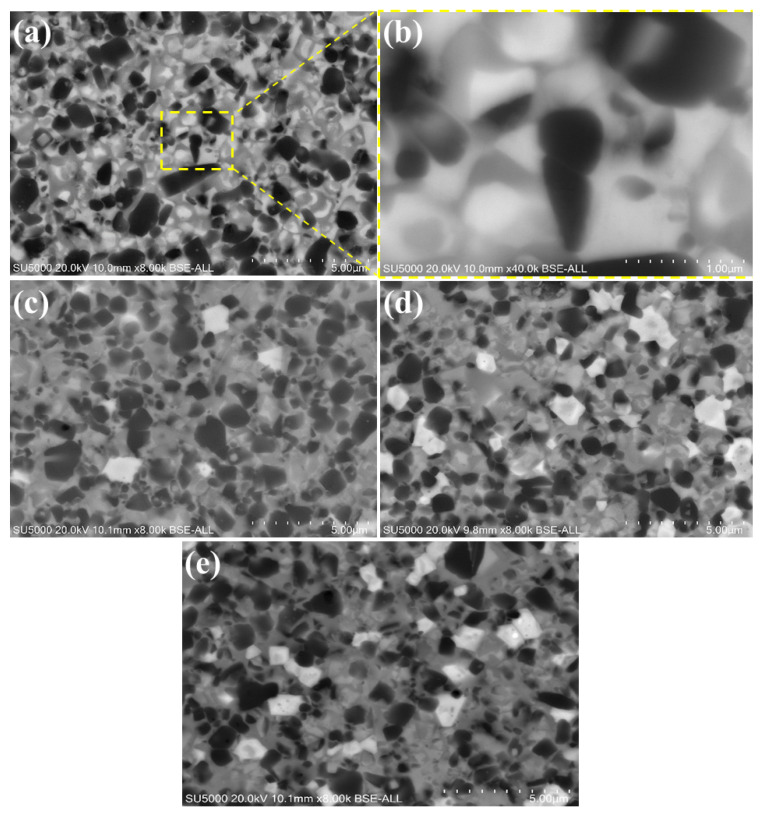
Scanning Electron Microscopy (SEM) images depicting Ti(C,N)-based cermets sintered with different contents of Si_3_N_4_: (**a**) Ti(C,N) + 0wt.%Si_3_N_4_, (**b**) enlarged view of Ti(C,N) + 0wt.%Si_3_N_4_, (**c**) Ti(C,N) + 2wt.%Si_3_N_4_, (**d**) Ti(C,N) + 4wt.%Si_3_N_4_, (**e**) Ti(C,N) + 6wt.%Si_3_N_4_.

Figure 5 shows the SEM images of the cermets sintered with various Si_3_N_4_ contents. All of the microstructures exhibit a typical black core having an inner white rim and a gray outside rim, as well as a gray core with a white rim structure. Ti(C,N) particles may dissolve during the sintering process and form a black core phase; however, the white core is the hard phase of the solid solution of Ti(C,N) particles with other dissolved additives [2]. As shown in Figure 5a, there are some grains with irregular and coarse shape. Elongated grains with a thinner adjacent grain boundary interface were observed in the specimen containing 0 wt.% of Si_3_N_4_ (Figure 5b). Si_3_N_4_ is recognized to prevent grain growth in cermets. In the absence of Si_3_N_4_, there is no inhibitory impact on grain growth, resulting in elongated grains. As seen in Figure 5c–e, with an increase in the content of silicon nitride, a new bright phase was formed. Furthermore, particles are more evenly distributed and have finer grains in Ti(C,N)-based cermets with 4 wt.% Si_3_N_4_. Nevertheless, as the fraction of silicon nitride grows, it consequently increases the proportion of solid solution of Ti(C,N) particles’ “white core”. The incorporation of Si_3_N_4_ to Ti(C,N)-based cermets may have a considerable impact on the particle size of the core phase and the rim thickness [41]. Raising the content percentage of silicon nitride in Ti(C,N)-based cermets up to 6 wt.% leads to the refinement of particle size, resulting in finer grains. In addition, it decreases the thickness of the rim in the cermet structure. The particle size of different phases is illustrated in Table 2. These variations in grain size and rim thickness may affect the mechanical and thermal properties of Ti(C,N)-based cermets and their performance during the cutting process, as will be detailed in the following Section 3.4 and Section 3.5.

### 3.4. Mechanical Properties

**Figure 6:**
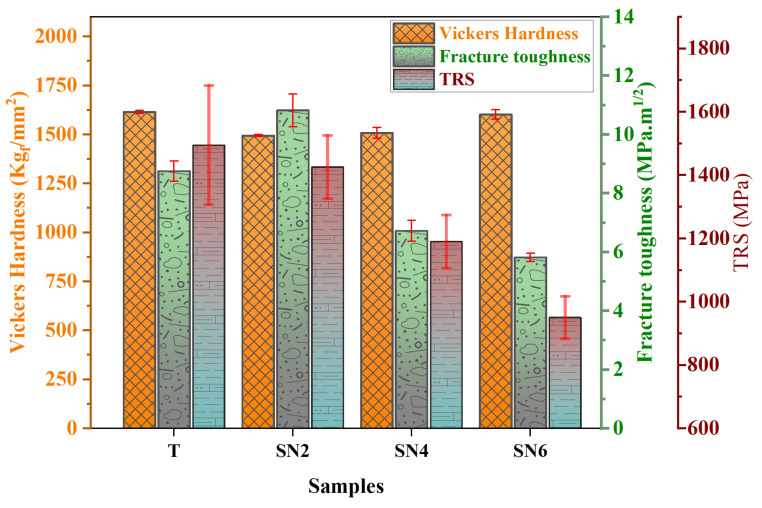
Mechanical properties of Ti(C,N)-based cermet with different Si_3_N_4_ contents. Error bars represent the standard deviation (SD).

**Figure 7:**
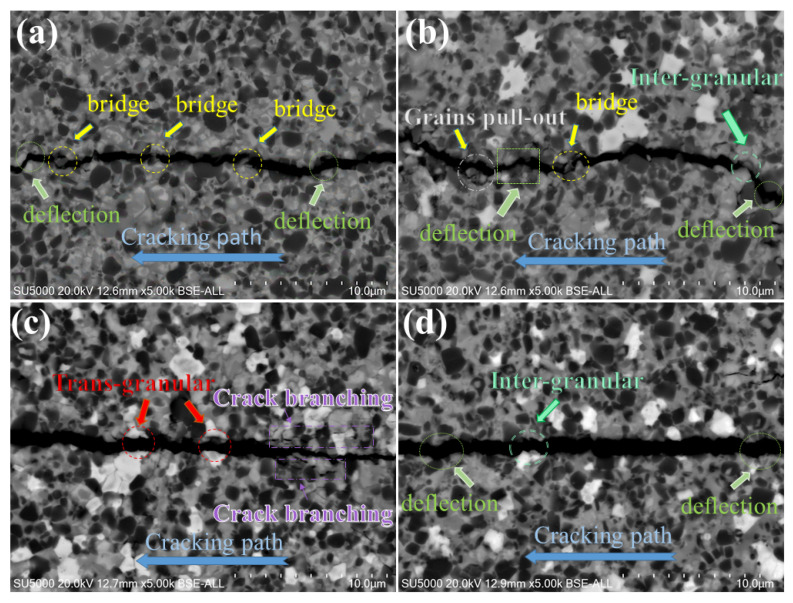
Crack propagation of Ti(C,N)-based cermets: (**a**) Ti(C,N) + 0wt.%Si_3_N_4_, (**b**) Ti(C,N) + 2wt.%Si_3_N_4_, (**c**) Ti(C,N) + 4wt.%Si_3_N_4_, (**d**) Ti(C,N) + 6wt.%Si_3_N_4_.

The effect of Si_3_N_4_ contents on the mechanical properties of the sintered Ti(C,N)-based cermet cutting tool is shown in Figure 6. The hardness property initially decreased slightly with the addition of silicon nitride to the Ti(C,N)-based cermets, followed by a slight increase again. After changing the content from 0 wt.% to 2 wt.%, the Vickers hardness value declined by 7.5% from 1615 to 1494 Kg_*f*_/mm^2^. However, the Vickers hardness increased again by 6.7% to reach 1602 Kg_*f*_/mm^2^ after increasing the content of Si_3_N_4_. Moving on, the fracture toughness rose from 8.75 to 10.82 MP${\mathrm{a.m}}^{1/2}$ after increasing the content of Si_3_N_4_. Nevertheless, fracture toughness fell from 10.82 to 5.82 MP${\mathrm{a.m}}^{1/2}$ after increasing the content of Si_3_N_4_. Varying amounts of Si_3_N_4_ additive in the cermets show a discernible pattern in the traverse rupture strength (TRS) measurements. The highest TRS of 1495.0 MPa is found in ceramics with 0 wt.% Si_3_N_4_, which indicates robust strength. Upon reaching 2 wt.% (SN2), the Si_3_N_4_ component causes a little drop in strength. However, certain SN2 samples exhibit higher TRS values than the T samples. Furthermore, a more notable decline in TRS is noted at a value of 1190.4 ± 84.0 MPa as the Si_3_N_4_ concentration approaches 4 wt.%. In conclusion, the materials containing 6 wt.% Si_3_N_4_ had the lowest TRS, measuring 950.6 ± 66.8 MPa, which suggests a notable reduction in strength when compared to the other compositions. The possible reason for the decrease in cermet bending strength may be attributed to the formation of the MSi phase (Section 3.2). The grain size of Ti(C,N)-based cermets has a major impact on their mechanical properties and overall performance, particularly Vickers hardness and fracture toughness. The specimens with smaller particles possess lower fracture toughness and a higher hardness, whereas coarse grains have a higher fracture toughness and a lower hardness [42]. Reduced distances between grain boundaries and the heightened effects of strengthening grain boundaries are two ways that smaller grain sizes in Ti(C,N) particles can lead to greater hardness. Nevertheless, extremely small grain sizes may result in a decrease in energy dissipation mechanisms and a higher possibility of intergranular fracture, which can diminish fracture toughness. The change in hardness values observed with the addition of Si_3_N_4_, which ranged from 0 wt.% to 6 wt.%, leads to increased fracture propagation resistance. Specifically, the greater hardness values of Ti(C,N)-based cermets with increased Si_3_N_4_ concentration may result in stronger microstructures, thereby impeding crack formation and propagation. Interestingly, the observed sequence is consistent with the fracture propagation behavior for the various specimens of Ti(C,N)-based cermets depicted in Figure 7. The formation and creation of the core/rim structure have a brilliant influence on the cracking path impedance during the indentation. The fracture toughness can be identified by the created crack path, which includes crack bridge, crack deflection, grain pull-out, crack branching, intergranular, and transgranular, by energy consumption [42]. With a change in the Si_3_N_4_ content, there is a relatively noticeable effect on the formed crack path. Because of the crack impedance in the Ti(C,N)+2 wt.% Si_3_N_4_ specimen, it promotes the highest fracture toughness of 10.82 MP${\mathrm{a.m}}^{1/2}$, as shown in Figure 7b. However, Ti(C,N)+6 wt.% Si_3_N_4_ samples have the lowest fracture toughness, which may be due to intergranular fracture during the crack path, demonstrating the relationship between microstructural modifications caused by Si_3_N_4_ addition and fracture toughness results.

### 3.5. Thermal Stability

**Figure 8:**
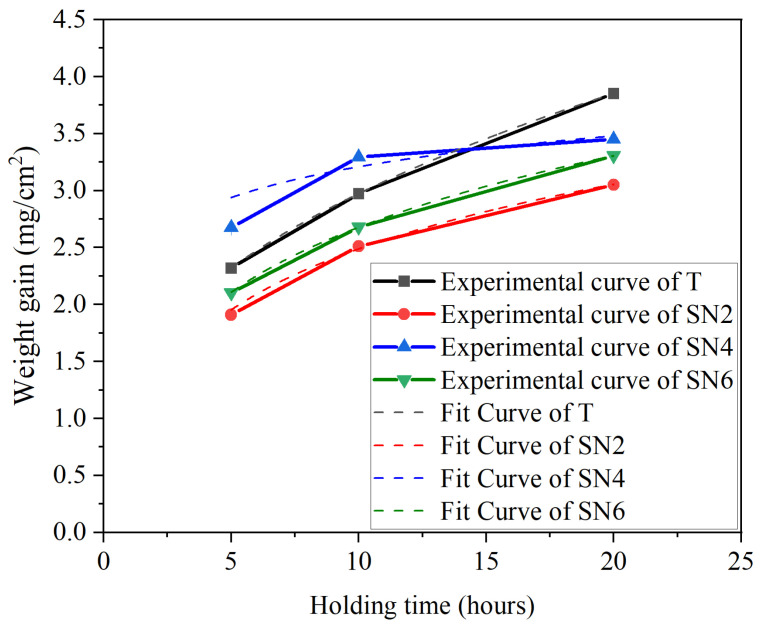
Mass gain of Ti(C,N)-based cermets after oxidation at 800 °C.

The formation of the oxide layer on Ti(C,N)-based cermets during oxidation is a critical factor influencing their behavior and properties. Initially, this process results in a rapid increase in weight. Figure 8 shows the weight gain per surface area (mg/cm^2^) in each Ti(C,N)-based cermet sample after isothermal oxidation treatment at 800 °C for 5, 10, and 20 h. After increasing the holding time of the oxidation process, the mass of Ti(C,N)-based cermet samples with varying Si_3_N_4_ contents is significantly affected. The curve of oxidation kinetics indicates that the weight increase in Ti(C,N)-based cermets followed a pattern of parabolic growth. Beginning with the holding time of 5 h, the mass of all samples increases sharply with about the same trend. As the holding time during the heating procedure increased from 5 to 20 h, the rate of mass growth of the samples increased slightly. Finally, for a 20 h holding time, almost all samples show an increase in mass gain. Among these, Ti(C,N)-based cermet samples with 0 wt.% Si_3_N_4_ content showed the highest weight gain. However, the rate of oxidation decreased over time for the Ti(C,N)-based cermet sample with a 4 wt.% Si_3_N_4_. During the early stages, rapid oxidation on the surface resulted in significant weight gain. Diffusion throughout the oxidation stage led to lower rates of weight gain over time. For the Ti(C,N)-based cermet sample with a 2 wt.%, the addition of Si_3_N_4_ exhibits optimum thermal stability that withstands a high heating temperature of 800 °C for 10 h. It can be explained as the formation of oxides; Si_3_N_4_ has the ability of thermal resistance [8,43]. Moreover, the relationship between the rate of weight gain and time can be modeled using the following equation [44]:

(5) $$\frac{\Delta m}{A_{s}}=K_{p}\times t^{n}+c$$

where Δ*m* represents the increase in mass, *A_s_* denotes the surface area, *K_p_* stands for the oxidation rate constant, *t* is the oxidation time, *n* represents the oxidation exponent, and *c* is a constant term. The results of the fitting process are illustrated in Figure 8. Table 3 displays the values of *K_p_*, *c*, and *n* for all the fitted curves, with the coefficient of determination (*R^2^*) indicating the quality of the fit.

**Figure 9:**
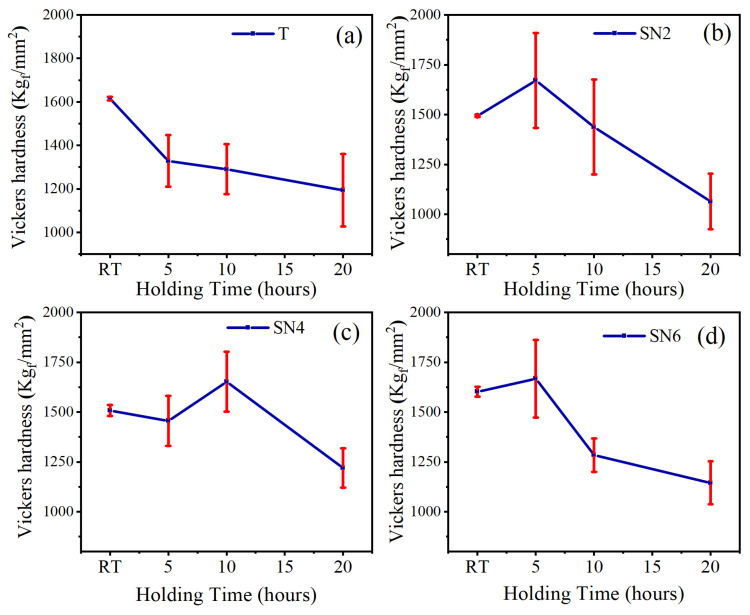
Vickers hardness of Ti(C,N)-based cermets after oxidation at 800 °C: (**a**) 0 wt.%Si_3_N_4_, (**b**) 2 wt.%Si_3_N_4_, (**c**) 4 wt.%Si_3_N_4_, (**d**) 6 wt.%Si_3_N_4_. Error bars represent the standard deviation (SD).

**Figure 10:**
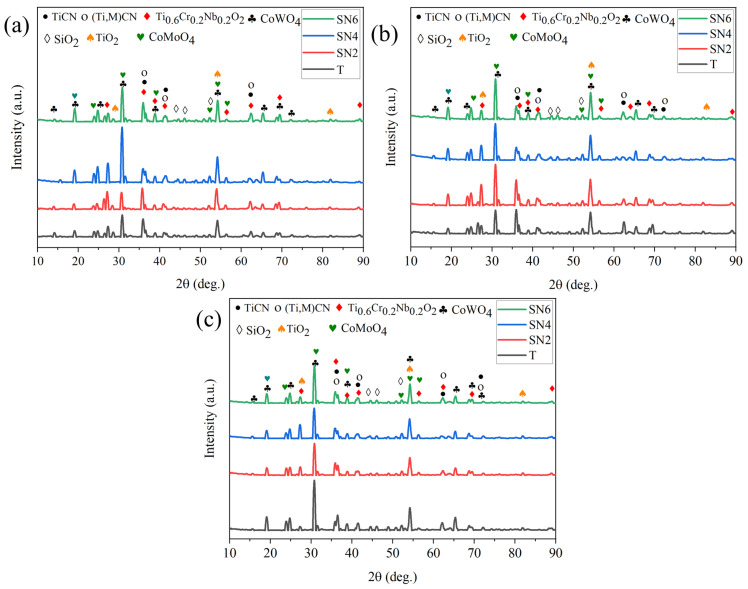
XRD of Ti(C,N)-based cermets after oxidation at 800 °C: (**a**) 5 h, (**b**) 10 h, (**c**) 20 h.

Figure 9 shows the effect of isothermal oxidation treatment on the hardness of Ti(C,N)-based cermet samples after heating at 800 °C for 5, 10, and 20 h. The hardness of Ti(C,N)-based cermets decreases with the increase in the holding time; the decline in hardness is due to grain growth and oxide layer formation [45,46]. However, hardness increased for samples of Ti(C,N)-based cermets with 2 wt.% wt. and 6 wt.% of Si_3_N_4_ for a holding time of 5 h and 4 wt.% of Si_3_N_4_ for a holding time of 10 h. The variations in properties occurs due to the formed oxides at the outer surface of Ti(C,N)-based cermet samples throughout the isothermal oxidation treatment process; the formed oxides at 5, 10, and 20 h are displayed in Figure 10. New oxide phases of Ti_0.6_Cr_0.2_Nb_0.2_O_2_, CoWO_4_, SiO_2_, TiO_2_, CoMoO_4_, and NiWO_4_ with JCPDS numbers 88-1983, 15-0867, 12-0708, 77-0443, 16-0309, and 72-0480, respectively, have developed with different diffraction angles. With an increase in the content of Si_3_N_4_ for each holding time, the overall diffraction peak shifts towards a lower angle side. This may be because of the formation of new phases with the addition of Si_3_N_4_. The lowest overall intensity peak occurs at 2 wt.% Si_3_N_4_ with 5 h holding time. However, with the increase in the holding time of isothermal oxidation treatment, the percentage of oxides increases relatively. Generally, Ti(C,N)-based cermet samples can withstand the oxidation process 800 °C up to 10 h. For the 20 h oxidation process, the formed oxides have the maximum intensity after the isothermal oxidation treatment process.

**Figure 11:**
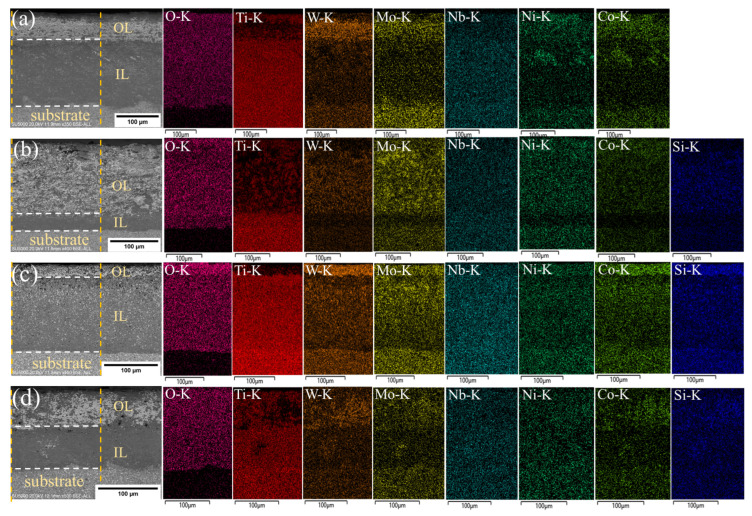
BSE/EDS patterns of cross-sections of Ti(C,N)-based cermets oxidized at 800 °C for 5 h with varying additions of Si_3_N_4_: (**a**) 0wt.%Si_3_N_4_, (**b**) 2wt.%Si_3_N_4_, (**c**) 4wt.%Si_3_N_4_, (**d**) 6wt.%Si_3_N_4_. The scanned area was between two orange dotted lines, and the oxide layers were separated by white dotted lines.

**Figure 12:**
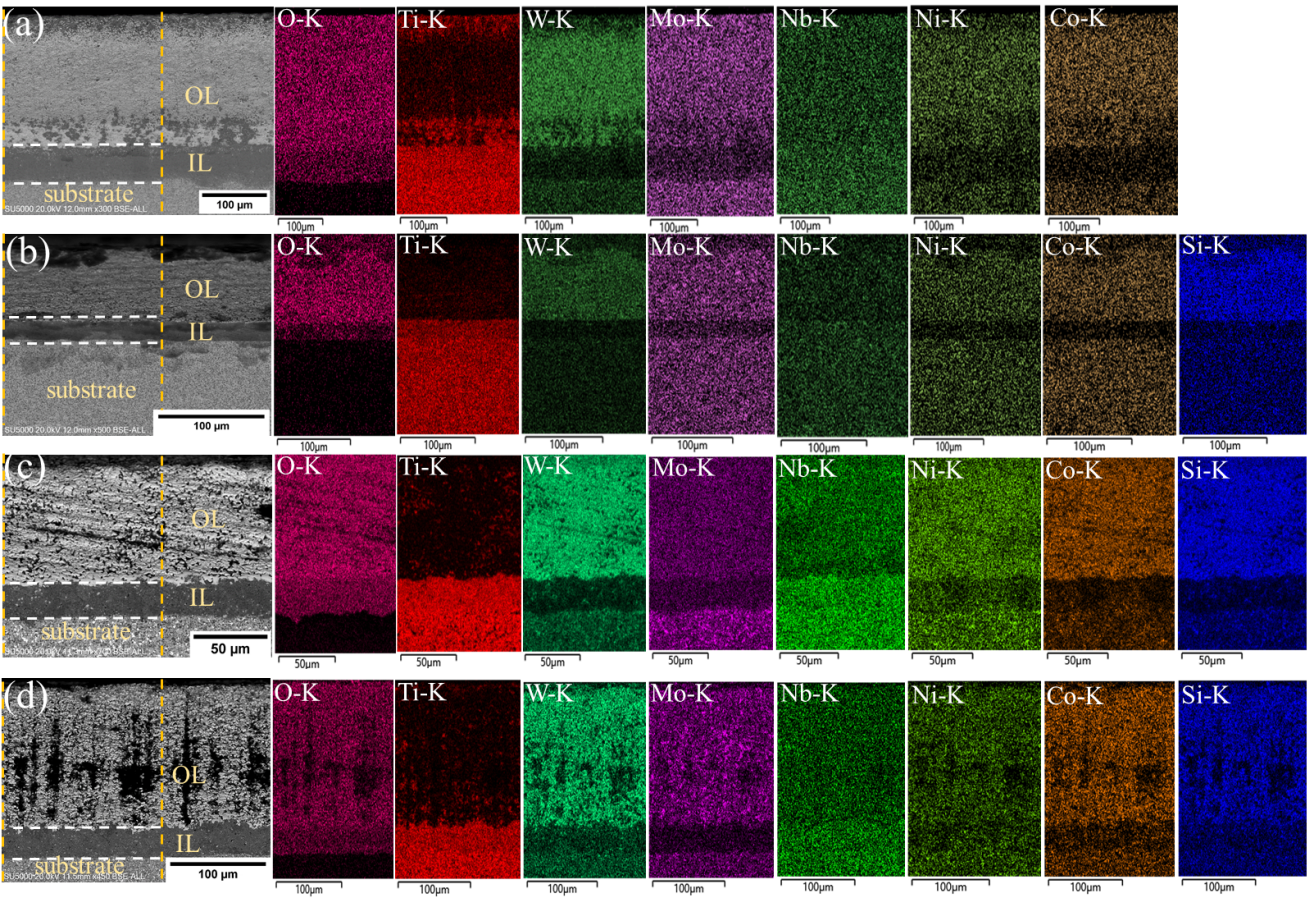
BSE/EDS patterns of cross-sections of Ti(C,N)-based cermets oxidized at 800 °C for 10 h with varying additions of Si_3_N_4_: (**a**) 0wt.%Si_3_N_4_, (**b**) 2wt.%Si_3_N_4_, (**c**) 4wt.%Si_3_N_4_, (**d**) 6wt.%Si_3_N_4_. The scanned area was between two orange dotted lines, and the oxide layers were separated by white dotted lines.

**Figure 13:**
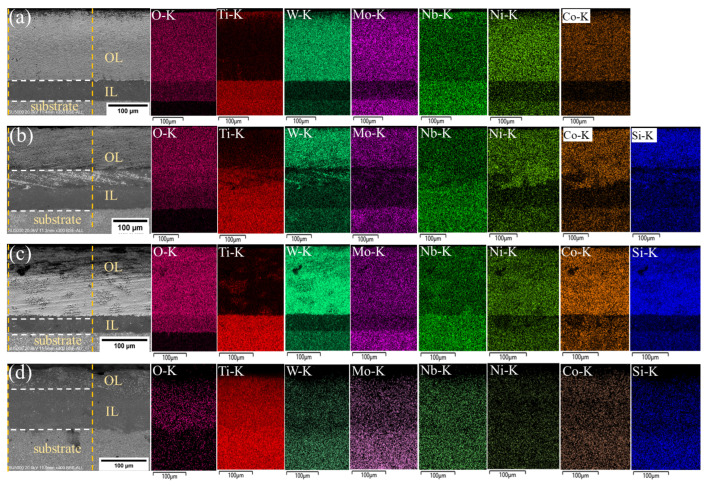
BSE/EDS patterns of cross-sections of Ti(C,N)-based cermets oxidized at 800 °C for 20 h with varying additions of Si_3_N_4_: (**a**) 0 wt.%Si_3_N_4_, (**b**) 2 wt.%Si_3_N_4_, (**c**) 4 wt.%Si_3_N_4_, (**d**) 6 wt.%Si_3_N_4_. The scanned area was between two orange dotted lines, and the oxide layers were separated by white dotted lines.

Backscattered electron (BSE) patterns and corresponding energy-dispersive X-ray spectroscopy (EDS) map scan results were utilized to investigate the microstructural development of Ti(C,N)-based cermets during oxidation at 800 °C for variable time periods of 5, 10, and 20 h with various amounts of Si_3_N_4_, as shown in Figure 11, Figure 12 and Figure 13. The scanned area was the region between two orange dotted lines. During oxidation, the cermet reacts with oxygen at elevated temperatures, leading to the formation of an oxide layer on its surface. Depending on the oxidation conditions, the oxide layer can take a variety of forms, including a single, continuous layer and several layers. In our experiment, BSE patterns predominantly comprise three regions: the substrate, intermediate layer (IL), and outer layer (OL). The duration of oxidation can affect the properties of the oxide layer, making it essential to understand this characteristic to enhance the oxidation resistance and mechanical properties of Ti(C,N)-based cermets for cutting applications.

Ti(C,N) particles were oxidized, producing a rutile phase (TiO_2_), where the Ti(C,N) surface cannot be entirely covered by the newly produced TiO_2_. As shown in the figures, the rutile phase mainly existed at the intermediate oxidized layer. The inclusion of different carbides (WC, Mo_2_C, and NbC) and alloying elements (Ni, Co) in the cermet composition might affect the oxidation behavior by changing the composition and structure of the oxide layer. Furthermore, the presence of Si_3_N_4_ may influence the oxidation kinetics and shape of the oxide layer. The EDS map displays the intensity of each element within the outer and intermediate oxide layers, while the oxides formed were previously displayed in Figure 10.

With increasing oxidation time, carbon may react with the additives, causing the formation of new phases, as shown in Figure 12c,d. One probable explanation for carbon formation at the oxide layer of the material is the existence of carbon-containing substances in the initially formed cermet composition, such as carbides (WC, Mo_2_C, and NbC) or organic binders. These substances that contain carbon may decompose during oxidation at high temperatures, releasing carbon atoms that may merge with oxygen to produce carbon dioxide (CO_2_) or carbon monoxide (CO). Silicon nitride is another factor to consider, as silicon can react with carbon to form silicon carbide (SiC), releasing additional carbon atoms that can contribute to the formation of carbon. Moreover, an increase in the Si_3_N_4_ content leads to a relatively higher proportion of the carbon phase (see Figure 12c,d). SiC is a stable compound that improves the oxidation resistance of cermets. It achieves this by producing an outer barrier of SiO_2_ and SiC on the surface, preventing the formation of the oxide layer. This layer protects the underlying material from additional oxidation and degradation, leading to enhanced thermal stability. Furthermore, the concentration of oxygen decreased from outer layer surface to substrate layer. The intermediate oxide layer frequently serves as a transition zone between the substrate and the outer oxide layer and has less chemical compositions than the other layer. Table 4 presents the average thickness of the oxide layer of Ti(C,N)-based cermets oxidized at 800 °C for 5, 10, and 20 h with varying additions of Si_3_N_4_. In summary, adding Si_3_N_4_ to Ti(C,N)-based cermets can greatly change their microstructure, leading to better mechanical properties, resistance to oxidation, thermal stability, and overall performance in applications with high demands. Thermal resilience of Ti(C,N)-based cermets allows experts to choose compositions suited for cutting operations, especially those that involve high temperatures generated between the interface of both workpiece and cutting tool.

## 4. Conclusions

In this study, Ti(C,N)-based cermets with various contents of Si_3_N_4_ were vacuum-sintered with a two-step reactive sintering process, and examinations of the microstructure, mechanical properties, and thermal stability show significant impacts from the addition of silicon nitride, offering practical benefits for cutting tool technology professionals. The outcome of this study can assist practicing professionals in achieving excellence in cutting tools by offering insights into material optimization and performance development. The following conclusions were obtained:

(1) The incorporation of Si_3_N_4_ to Ti(C,N)-based cermets enhances the quantity of white core solid solution and has a significant influence on the particle size of the core phase and rim thickness. As the content of silicon nitride in Ti(C,N)-based cermets is increased to 6 wt.%, it causes a refinement in particle size and generates finer grains. Furthermore, it simultaneously reduces the thickness of the rim within the structure of the cermet. These microstructural changes can help experts produce cutting tools with enhanced material quality.
(2) The mechanical properties of Ti(C,N)-based cermets were affected by varying the contents of Si_3_N_4_, where the mass, hardness, and fracture toughness of oxidized samples were influenced. Experimentally, Ti(C,N)-based cermet samples with 2% Si_3_N_4_ exhibited excellent mechanical properties including Vickers hardness (1494 Kg_*f*_/mm^2^), traverse rupture strength (1425.8 MPa), and fracture toughness (10.82 MP${\mathrm{a.m}}^{1/2}$). However, Ti(C,N)-based cermet samples with 4 wt.% Si_3_N_4_ exhibit a Vickers hardness of 1602 Kg_*f*_/mm^2^ and fracture toughness of 6.70 MP${\mathrm{a.m}}^{1/2}$. These findings enable professionals to pick the suitable Si_3_N_4_ content for certain mechanical performance objectives.
(3) The thermal stability of Ti(C,N)-based cermets was enhanced with the addition of Si_3_N_4_ because of its thermal resistance; in addition, the mass and Vickers hardness are changed with its inclusion. For the 5 h isothermal oxidation process at 800 °C, Ti(C,N)-based cermets with 2 wt.% and 6 wt.% will be the best choice of relative oxidation resistance. This information is critical for manufacturing cutting tools that can operate in high-temperature conditions.

## Figures and Tables

**Table 1:** Initial powder compositions in weight percentages for various cermet preparations.

Specimen	Ti(C,N)	$\begin{array}{c} \mathrm{WC}\;+\;{\mathrm{Mo}}_{2}C\\ \left(1:1\right) \end{array}$	NbC	$\begin{array}{c} \mathrm{VC}\;+\;{\mathrm{Cr}}_{3}C_{2}\\ \left(1:1\right) \end{array}$	$\begin{array}{c} \mathrm{Ni}\text{-}\mathrm{Co}\\ \left(1:1\right) \end{array}$	Si_3_N_4_
T	58	20	6	1	15	0
SN2	56	20	6	1	15	2
SN4	54	20	6	1	15	4
SN6	52	20	6	1	15	6

**Table 2:** Thickness of black core, white core, and rims of Ti(C,N)-based cermets ^1^.

Phase	T	SN2	SN4	SN6
Black core	0.59 ± 0.27	0.591 ± 0.22	0.512 ± 0.216	0.444 ± 0.222
White rim	0.085 ± 0.025	0.050 ± 0.023	0.056 ± 0.026	0.066 ± 0.031
Grey rim	0.203 ± 0.06	0.163 ± 0.045	0.1402 ± 0.043	0.129 ± 0.043
White core	0.48 ± 0.19	0.457 ± 0.158	0.466 ± 0.167	0.431 ± 0.173
Grey rim	0.148 ± 0.038	0.189 ± 0.061	0.175 ± 0.055	0.173 ± 0.053

^1^ Dimension in μm.

**Table 3:** The values of oxidation rate (*K_p_*), the oxidation exponent (*n*), constant term (*c*), and the coefficient of determination (*R^2^*) calculated by fitting the curves in Figure 8 using Equation (5).

Cermets	*K_p_*	*n*	*C*	*R*^2^ ^1^
T	1.28 ± 0.018	0.367 ± 0.005	3.337 × 10^−4^ ± 0.013	0.99994
SN2	1.148 ± 0.116	0.329 ± 0.034	−0.0016 ± 0.078	0.99657
SN4	2.095 ± 0.402	0.174 ± 0.069	−0.0012 ± 0.209	0.9834
SN6	1.26 ± 0.055	0.323 ± 0.015	−7.36 × 10^−4^ ± 0.036	0.99936

^1^ An indicator of the quality of fit.

**Table 4:** The average thickness of the oxide layer of Ti(C,N)-based cermets at 800 °C for 5, 10, and 20 h ^1^.

Cermets	5 h	10 h	20 h
T	210.52 ± 5.3	235.45 ± 7.4	245.75 ± 4.52
SN2	140.06 ± 8.5	160.73 ± 4.7	210.23 ± 3.7
SN4	115.24 ± 6.2	120.43 ± 3.4	150.58 ± 5.3
SN6	132.64 ± 4.3	165.28 ± 6.3	170.92 ± 4.6

^1^ Dimension in μm.

## Data Availability

The data presented in this study are available on request from the corresponding author. The data are not publicly available due to privacy.

## Abbreviations

The following abbreviations are used in this manuscript:

SPS	Spark Plasma Sintering
PEG	Polyethylene Glycol
HP	Hot pressing
MS	Microwave Sintering
HIP	Hot Isostatic Pressing
Ti(C,N)	Titanium carbonitride
XRD	X-Ray Diffraction
SEM	Scanning Electron Microscope
EDS	Energy Dispersive X-ray Spectroscopy
SD	Standard Deviation
TRS	Traverse Rupture Strength
IL	intermediate layer
OL	Outer layer
